# Effect of Temperature on the Development and Survival of *Feltiella acarisuga* (Vallot) (Diptera: Cecidomyiidae) Preying on *Tetranychus urticae* (Koch) (Acari: Tetranychidae)

**DOI:** 10.3390/insects12060508

**Published:** 2021-05-31

**Authors:** Yong-Seok Choi, Sunghoon Baek, Min-Jung Kim

**Affiliations:** 1 Bioenvironmental Division, Chungnam Agricultural Research and Extension Services, Yesan 32418, Korea; yschoi92@korea.kr (Y.-S.C.); shbaek007@hotmail.com (S.B.); 2 Department of Industrial Entomology, Korea National College of Agriculture and Fisheries, Jeonju 54874, Korea; 3 Department of Plant Medicals, Andong National University, Andong 36729, Korea

**Keywords:** *Feltiella acarisuga*, predatory gall midge, thermal threshold, development rate, survivorship, mass rearing, biological control

## Abstract

**Simple Summary:**

The predatory gall midge is one of the most effective natural enemies of the two-spotted spider mite, which is an economic pest causing a severe impact on a wide variety of plants worldwide. In general biocontrol programs, midges have been reared in insectaries and released into agriculture fields or greenhouses. In this study, we tested the effect of temperature on the development and survival of the predatory gall midge. The results showed that temperature played a crucial role in determining the emergence time and survival rate at each development stage. We described the responses to temperatures using several equations and estimated the values for explaining the observed biological patterns. The developed equations suggested that 23.3–28.7 °C temperatures were suitable for the total immature stage. In contrast, conditions around 8 °C and 35 °C should be avoided due to the high mortality. These results can help to determine the effective temperatures for both rearing and releasing, and ultimately contribute to the successful biological control of the spider mite.

**Abstract:**

The predatory gall midge, *Feltiella acarisuga* (Vallot) (Diptera: Cecidomyiidae), is an acarivorous species that mainly feeds on spider mites (Acarina: Tetranychidae). Because of its cosmopolitan distribution and predation efficacy, it is considered an important natural enemy available as a biological agent for augmentative biocontrol. However, despite its practical use, the thermal development and survival response to temperature have not yet been fully studied. In this study, we investigated the stage-specific development and survival of *F. acarisuga* at seven temperatures (11.5, 15.7, 19.8, 23.4, 27.7, 31.9, and 35.4 °C) to examine the effect of temperature on its lifecycle. All developmental stages could develop at 11.5–31.9 °C, but the performance was different according to the temperature. From the linear development rate models, the lower development threshold and thermal constant of the total immature stage were estimated at 8.2 °C and 200 DD, respectively. The potential optimal and upper threshold temperatures for the total immature stage were estimated as 29.3 and 35.1 °C using a non-linear development model. The operative thermal ranges for development and survival at 80% of the maximum rate were 24.5–32.3 and 14.7–28.7 °C, respectively. Thus, it was suggested that 24.5–28.7 °C was suitable for the total immature stage. In contrast, conditions around 8 °C and 35 °C should be avoided due to the lower development rate and high mortality. Our findings provide fundamental information for an effective mass-rearing and releasing program of *F. acarisuga* in an augmentative biocontrol program and help to predict phenology.

## 1. Introduction

The two-spotted spider mite, *Tetranychus urticae* (Koch, 1836) (Acari: Tetranychidae), is a highly polyphagous pest, which causes significant yield losses in agricultural crops such as strawberries, tomatoes, grapes, pears, and apples [1,2,3]. Plant leaves infested with *T. urticae* undergo a decrease in photosynthesis, and even defoliation when the damage is severe [4]. Webbing and staining on the plant body can also lead to reductions in the commercial value of crops [5]. The application of synthetic acaricides has been a common method for controlling this pest [2]. However, it has caused the serious problem of insecticide resistance due to the high reproductive potential and short lifecycle of spider mites in a limited space, such as greenhouse conditions [4,6]. Moreover, insecticides have an undesirable impact on non-targeted organisms including natural enemies of the spider mite and the natural environment [7]. Biological control is an alternative that can reduce insecticide input and increase the success of integraded pest management (IPM) programs. Phytoseiidae mites such as *Phytoseiulus persimilis* (Athias-Henriot, 1957), *Neoseiulus californicus* (McGregor, 1954), *Amblyseius womersleyi* (Schicha, 1975), and some predacious Cecidomyiidae, have been mainly used as biological agents for controlling spider mites [2,3,8,9,10].

The predatory gall midge, *Feltiella acarisuga* (Vallot, 1827) (Diptera: Cecidomyiidae), is an acarivorous species, which mainly feeds on spider mites (Acarina: Tetranychidae) [8,11,12]. This insect distributes worldwide, except for south America [9,10,13], and thus, is considered an important natural enemy available as a biological agent for controlling spider mites [8,11,12]. *Feltiella acarisuga* naturally occurs on plants highly infested with *T. urticae* in various crop systems. The adult female lays about 30 eggs during her lifespan in areas of high prey density. The emerging larvae feed on an average of 30 mixed stages of *T. urticae* per day and form a cocoon after three larval stages. *Feltiella acarisuga* is more predacious and distinctly mobile than the predatory mites commonly used for the biological control of *T. urticae* [9,12,14,15]. Because of these characteristics, *F. acarisuga* has been widely used in augmentative biological control (ABC) programs as a part of IPM, in which mass-reared predators are released [16]. It is known that the release of 1000 individuals per hectare is effective in controlling *T. urticae* on tomato, pepper, and cucumber crops. It is also applied to greenhouse vegetable crops such as strawberries [9].

Temperature is one of the key factors affecting an insect’s emergence and survival because its developmental performance varies according to temperature, and it can develop within a unique limited temperature range [17]. In general, these developmental characteristics have been expressed as a unimodal curve of the relationship between developmental performance and temperature [18]. Thus, the optimal range showing good performance of the target insect can be estimated through the development curve. Understanding the thermal development of biological agents is important for effective mass-rearing. Moreover, studies on the developmental response of *F. acarisuga* according to temperature can provide information for releasing programs. For example, the appropriate release timing for successful establishment can be determined based on the parameters of temperature-dependent development [19].

Several studies have addressed the demographic parameters of *F. acarisuga*, including predation, longevity, and fecundity in some constant temperature conditions [12,14,20,21,22,23]. These studies are very useful for evaluating the performance of biological agents associated with sustainability and predation effectiveness in specific releasing conditions. However, the development of *F. acarisuga* over the entire thermal range (i.e., including both ends of the potential extremes) is not well documented. Gillespie et al. [24] first reported the temperature and relative humidity (RH) effects on the development of *F. acarisuga*. That study estimated the threshold temperature (°C) for development (i.e., lower developmental threshold) through an experiment at four temperatures. However, an extreme temperature range was not examined. Kim et al. [23] also reported thermal development in mid-range temperatures. Other studies examined the development time at specific single constant temperatures [14,25]. Although those studies are valuable, they are insufficient to describe the detailed developmental characteristics, including the optimal and operative range temperatures. Therefore, the objectives of this study were to (1) determine the stage-specific development of *F. acarisuga* at various temperatures incorporating potential extreme temperatures, (2) develop a temperature-dependent development and survival model of *F. acarisuga*, and (3) estimate the biological parameters of *F. acarisuga* supporting an effective ABC program.

## 2. Materials and Methods

### 2.1. Insects and Their Preys

A laboratory colony of *F. acarisuga* was used in this study. The colony started from larvae collected in papaya (*Carica papaya* var. Red Lady) greenhouse in the Chungcheongnam-do Agricultural Research and Extension Services (36°44′31.9″ N, 126°49′12.3″ E) on 11 July 2019. The collected larvae were reared on kidney bean (*Phaseolus vulgaris*) plants infested with *T. urticae*. The infested kidney bean plants were provided every two days. For rearing the mite prey, fresh plants (<two weeks old) were provided at two-day intervals. The *F. acarisuga* colony and their prey mites were maintained in acryl cages (50 × 30 × 40 m) in a breeding room under constant temperature (26 ± 0.5 °C) with 70% relative humidity (RH) with a 14:10 (L:D) photoperiod.

### 2.2. Development Experiment

To examine the effect of temperature on the development of *F. acarisuga*, its immature stages were exposed to seven different temperatures (11.5, 15.7, 19.8, 23.4, 27.7, 31.9, and 35.4 °C) with approximately 90% (RH) and a 14:10 (L:D) photoperiod in a growth chamber (JSMI-05CL, JSR; Gongju, Korea). The experimental temperatures were chosen at 4 °C intervals. During the experiment, the inside temperature and RH of each growth chamber were monitored using a data logger (H08-007-02, OnSet Computer Corp; Pocasset, MA, USA).

The cocoons were separated from the laboratory colony of *F. acarisuga* into an acryl cage under the breeding room conditions previously described to obtain an experimental egg cohort. Kidney bean pots infested with *T. urticae* were prepared in the cage and replaced every day. The plants were sprayed with a 50% sugar solution (*w*/*v*) to promote the oviposition of the adults [26]. Then, the adults emerging from the cocoons were allowed to lay their eggs on the bean leaves for 24 h. Because it is not easy to detach individual eggs from the leaves without injury, we first collected the whole leaves. The leaves were cut into the form of leaf discs (3 cm in diameter) to count the eggs accurately and quickly while minimizing exposure to room temperature. The leaf discs were then separately transferred to a Petri dish with a pre-loaded absorbent pad (47 mm in diameter) (1217M59, Merck Millipore, MA, USA). Before placing the leaf discs, the pad in the Petri dish was fully soaked with tap water, and the dishes were allocated into chambers at seven different temperatures. Egg hatching was checked daily, and the development time and survival rate of the eggs were calculated.

To obtain a large enough sample size for the experiments after the egg stages, we prepared another preliminary egg cohort (<24 h old) produced in the breeding room conditions. These eggs were reared using the same method as previously described at seven different temperature conditions, but the survival rates were not determined. The first instar larvae (<24 h old) from both egg experiments and the preliminary rearing colony were treated at the same temperature they experienced in the egg stages except for 35.4 °C. Because no eggs hatched at 35.4 °C in this study, the first instar larvae emerging at 27.7 °C were used to test larval development at 35.4 °C. The newly emerging first instar larvae were separately transferred to a leaf disc infested with *T. urticae* and moved into Petri dishes with an absorbent pad soaked with water. More than 70 larvae at each temperature were prepared (Table 1; one individual at 19.8 °C was missing during the experiment). For larvae development, food was provided at two-day intervals as a leaf disc from a laboratory mite culture until cocoon formation, and water was supplied every day. Each Petri dish was checked daily to determine whether pupation and further emergence to adults was proceeding. In our experiment, larvae also were not developed to pupae at 35.4 °C; newly formed pupae from the breeding room conditions were used to estimate the pupal survival rate and development time at 35.4 °C (Table 1).

### 2.3. Data Analysis and Model Development

- Effect of temperature on development time

The effect of temperature on the development time (days) in each developmental stage was evaluated using analysis of variance (ANOVA), followed by a post hoc comparison using Tukey’s honest significant difference (HSD) test at a 5% error rate.

- Development rate model

The development rates (1/day) were calculated as the reciprocal values of the mean development time for further analyses. The rates were fitted against the temperature (°C) by the linear model and non-linear model. Each developmental stage (egg, larval, and pupal) and the total immature stage (egg to adult emergence) were fitted, respectively. The parameters of linear model were estimated using PROC REG in SAS 9.4 [27]. The linear portion of the developmental rates was used in line fitting [28]:

(1) $$r\left(T\right)=aT+b$$

where $r\left(T\right)$ is the development rate at a certain temperature T °C, and a and b are the slope and intercept of the linear equation, respectively. The lower developmental threshold was estimated as −b/a ([29]), and the thermal constant (degree days, DD) was estimated as 1/a [30].

To estimate the “conceptual” upper development thresholds ($T_{2}$) and operative thermal range by incorporating the non-linear portion of the development rates, we used the following six models (i-vi). The observed developmental rate was fitted against experimental temperature, using PROC NLIN in SAS 9.4 [27]. Due to the risk of bias estimation of the upper threshold ($T_{2})$, we excluded the data point at 35.4 °C that all individuals did not develop to next stage from curve fitting (Table 1).

(i) Lobry–Rosso–Flandrois (LRF) model [31,32]:

(2) $$r\left(T\right)=\mu_{opt}\frac{\left(T-T_{2}\right){\left(T-T_{1}\right)}^{2}}{\left(T_{opt}-T_{1}\right)\left[\left(T_{opt}-T_{1}\right)\left(T-T_{opt}\right)-\left(T_{opt}-T_{2}\right)\left(T_{opt}+T_{1}-2T\right)\right]}$$

where $r\left(T\right)$ is the development rate at a certain temperature T °C, and $\mu_{opt}$ is development rate at $T_{opt}$ (°C). $T_{1}$ and $T_{2}$ represents lower and upper development thresholds at which the development rate is conceptually zero in LRF model.

(ii) Logan-6 model [33]:

(3) $$r\left(T\right)=\psi \left[Exp\left(\rho T\right)-Exp\left(\rho T_{u}-\frac{T_{u}-T}{\Delta T}\right)\;\right]$$

where ψ, ρ, $T_{u}$, and ΔT are parameters to be estimated. $T_{u}$ is the upper lethal temperature, and ΔT is the temperature range over which physiological breakdown becomes the overriding influence.

(iii) Lactin-2 model [34]:

(4) $$r\left(T\right)=Exp\left(\rho T\right)-Exp\left(\rho T_{u}-\frac{T_{u}-T}{\Delta T}\right)+\lambda$$

In the Lactin-2 model, the parameters ρ, $T_{u}$, and ΔT have same meaning as in the Logan-6 model. λ is a constant that leads the curve to intersect with the temperature-axis in suboptimal range (i.e., lower development threshold).

(iv) Performance-2 model [35,36,37]:

(5) $$r\left(T\right)=c\left(T-T_{1}\right)(1-Exp\left[K\left(T-T_{2}\right)\right]$$

where c and K are parameters to be estimated. The Performance-2 model is a simplified equation of Performance-1 model and its square-rood form is Ratkowsky model.

(v) Beta model [38,39]:

(6) $$r\left(T\right)=r_{m}\left(\frac{T_{2}-T}{T_{2}-T_{m}}\right){\left(\frac{T-T_{1}}{T_{m}-T_{1}}\right)}^{\frac{T_{m}-T_{1}}{T_{2}-T_{m}}}$$

where $r_{m}$ is a parameter of the maximum development rate at optimum development temperature ($T_{m}$).

(vi) Briere-2 model [18]:

(7) $$r\left(T\right)=\alpha T\left(T-T_{1}\right){\left(T_{2}-T\right)}^{1/\beta }$$

where α is an empirical constant to be estimated, and β is a parameter determining shape of curve in high temperature range.

The conformance of six non-linear models was evaluated based on the goodness of fit to observed data and whether the potential upper limit temperature could be described by models. Since the examined models all have four parameters, the goodness of fit was assessed by the residual sum of squares (RSS), not considering structural complexity of models. The data point at 35.4 °C, which is not included in the curve fitting, was also used in RSS calculation.

(8) $$RSS=\overset{n}{\underset{i=1}{\sum }}{\left(r_{i}-{\hat{r}}_{i}\right)}^{2}$$

where n is the sample size, $r_{i}$ is the i-th observation of development rate at a certain experimental temperature, and ${\hat{r}}_{i}$ is the predicted rate from each model. Then, the conceptual upper threshold ($T_{2}$) from each model was compared to the two experimental temperatures of both 31.9 and 35.4 °C expected to be closest to the upper limit in our experiment. In the Logan-6 and Lactin-2 equations, the conceptual upper threshold ($T_{2}$) was obtained by numerical approach (i.e., where $r\left(T\right)=0$ in the high temperature range). We classified the model results to three categories: 1. acceptable (31.9 °C < $T_{2}$ ≤ 35.4 °C), 2. suspicious (35.4 °C < $T_{2}$ < 37 °C), and 3. unreliable ($T_{2}$ ≤ 31.9 or 37 °C ≤ $T_{2}$). From these procedures, we finally selected a model showing good performance in both RSS and upper threshold in describing all development stages. The operative thermal range ($B_{80}$) showing 80% of the maximum rate was computed based on selected final models [40].

- Distribution of development completion

The variation in development time for each stage was described using the cumulative frequency distribution of developmental completion. The development times were normalized to the median development time to characterize the temperature-independent development distribution [41]. The cumulative frequency of development was then fitted against the normalized time (day/median day) using the two-parameter Weibull cumulative function [41]. The data for all temperatures of each stage were used to fit the model after normalizing the development time.

(9) $$F\left(x\right)=100-100\left\{1-Exp\left[{\left(-x/\alpha \right)}^{\beta }\right]\right\}$$

where $F\left(x\right)$ is the cumulative frequency (%) of development completion at a certain developmental stage at normalized time x, α is a shape parameter, and β is a parameter. The development completion model was fitted using PROC NLIN in SAS 9.4 [27].

- Effect of temperature on survival

In each egg, larval, and pupal stage, the survival rate was calculated by dividing the number of individuals that successfully developed into the next stage by the initial number at a specific stage. The survival rate of the total immature period was calculated by multiplying the survival rates from larvae to adult emergence by the survival rate of the eggs. The survival rates of the eggs and pupae showed relatively high survival rates at 11.5 °C, which is the lowest temperature in our experiments (Table 1). Because the survival curve is generally a symmetric bell-shaped distribution [42], we used imaginal points, the lower development thresholds of egg and pupa, calculated from each of linear models. Each stage-specific survival was fitted against the temperature using the following model [42]:

(10) $$S\left(T\right)=100-Exp\left[a+bT+cT^{2}\right]$$

where $S\left(T\right)$ is the survival rate (%) at a certain temperature T °C, and a, b, and c are parameters to be estimated. The parameters of the temperature-dependent survival model were estimated using PROC NLIN in SAS 9.4 [27]. From the developed model, the operative thermal range ($B_{80}$) showing 80% of the maximum survival rate was determined [40].

## 3. Results

The development time of *F. acarisuga* eggs was significantly affected by temperature (*F* = 543.7; df = 5, 379; *p* < 0.001). The temperature effect on larva and pupal development was also significant (larva: *F* = 181.8; df = 5, 219; *p* < 0.001; pupa: *F* = 327.4; df = 5, 173; *p* < 0.001; total immature: *F* = 557.3; df = 5, 173; *p* < 0.001). From 11.5 to 31.9 °C, *F. acarisuga* could develop from eggs to adults, but the survival varied according to the developmental stage and temperature regime (Table 1). All immature stages could not completely transfer to the next stage at 35.4 °C. Some pupae developed at 35.4 °C, but they eventually died during eclosion. The longest development time of any of the immature stages was in the larval stage at all temperature ranges.

**Figure 1:**
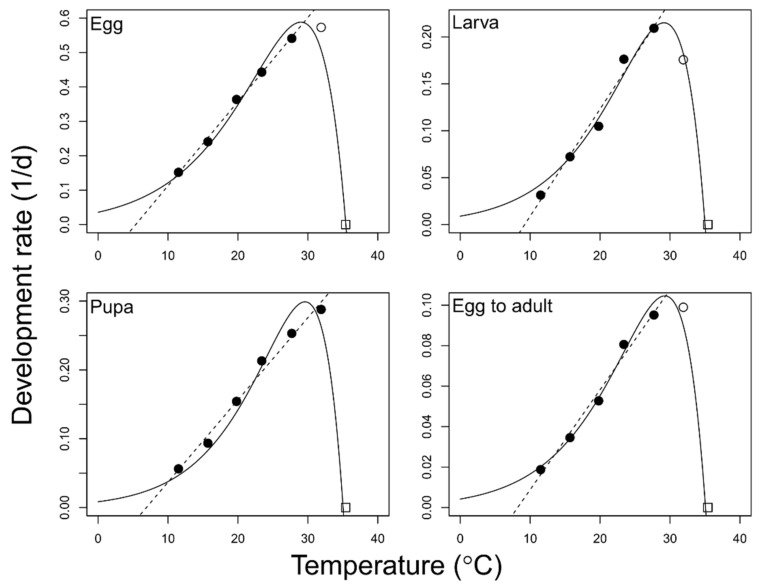
Temperature-dependent development rate models for *Feltiella acarisuga*. Linear (dashed lines) and non-linear Logan-6 (solid lines) models. Open circle data points were excluded in the linear regression analysis. All data except for open square data points (35.4 °C) were used in the curve fitting.

The linear portions of the development rates (1/development time) were well explained by the linear model at all developmental stages (egg: *F* = 1004.0; df = 1, 3; *p* < 0.001; *r*^2^ = 0.997; larva: *F* = 126.5; df = 1, 3; *p* < 0.05; *r*^2^ = 0.977; pupa: *F* = 309.3; df = 1,4; *p* < 0.001; *r*^2^ = 0.987; total immature: *F* = 183.3; df = 1, 3; *p* < 0.001; *r*^2^ = 0.984) (Figure 1 and Table 2). The lower developmental thresholds for the egg, larval, pupal, and total immature stages were calculated to be 5.5, 9.5, 6.9, and 8.2 °C, respectively. The thermal constants required to complete the development of the egg, larval, pupal, and total immature stages were 41.7, 90.9, 83.3, and 200.0 DD, respectively.

Among the six non-linear models, Logan-6 showed best fit and reliable parameter value of conceptual upper threshold in all development stages (Table 3). The development rates over the entire temperature range incorporating the non-linear portion for all stages were well described by the Logan-6 model (egg: *F* = 76.09; df = 4, 2; *p* < 0.05; larva: *F* = 119.81; df = 4, 2; *p* < 0.001; pupa: *F* = 53.23; df = 4, 2; *p* < 0.05; total immature; *F* = 104.37; df = 4, 2; *p* < 0.05) (Figure 1 and Table 4). The optimum temperature ($T_{opt}$) showing the maximum development rate for the egg, larval, pupal, and total immature stages was estimated to be 29.0, 29.1, 29.6, and 29.3 °C, with development rates of 0.588, 0.215, 0.299 and 0.105, respectively. The 80% operative thermal range ($B_{80}$) for the egg, larval, pupal, and total immature stages were 23.7–32.4, 24.3–32.2, 25.1–32.5, and 24.5–32.3 °C, respectively.

**Figure 2:**
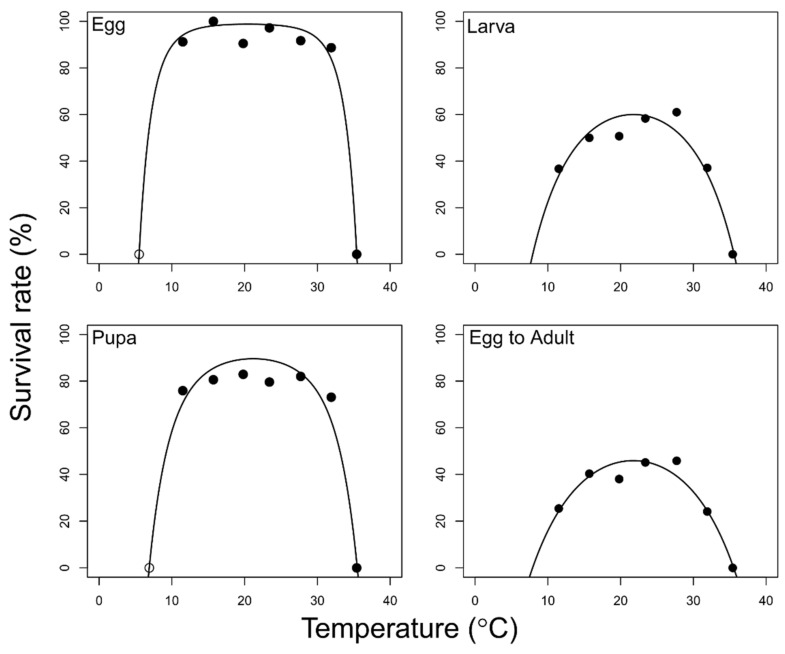
Temperature-dependent survival models (solid lines) for *Feltiella acarisuga*. Open circle data points are not experimental data but imaginal points, the lower development thresholds.

**Figure 3:**
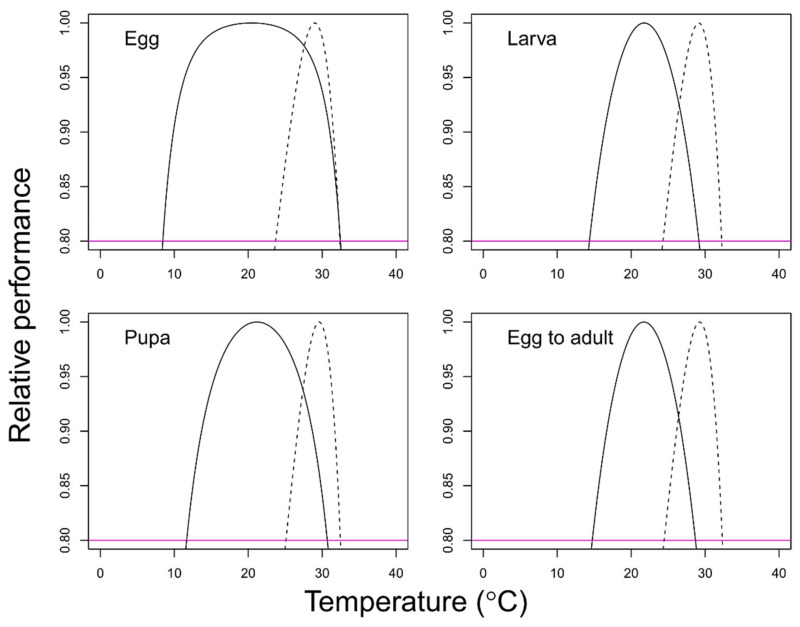
Temperature range expecting to show relatively good performance (> 80% of the maximum survival and development rate) for the development rates and survival of *Feltiella acarisuga*. Survival rate (solid lines) and development rate (dashed lines) models.

The temperature-dependent survival models provided good fit for each developmental stage (egg: *F* = 671.63; df = 3, 5; *p* < 0.001; larva: *F* = 114.83; df = 3, 4; *p* < 0.001; pupa: *F* = 196.43; df = 3, 5; *p* < 0.001; total immature; *F* = 112.2; df = 3, 4; *p* < 0.001) (Figure 2 and Table 5). The $B_{80}$ range for the survival rates was estimated as 9.7–32.7 (>79.1%), 14.4–29.2 (>48.0%), 11.7–30.7 (>71.7%), and 14.7–28.7 (>36.7%) for the egg, larval, pupal, and total immature stages, respectively. Both the $B_{80}$ values for temperature-dependent survival and the non-linear development model were plotted to visualize the optimal operating temperature (Figure 3).

**Figure 4:**
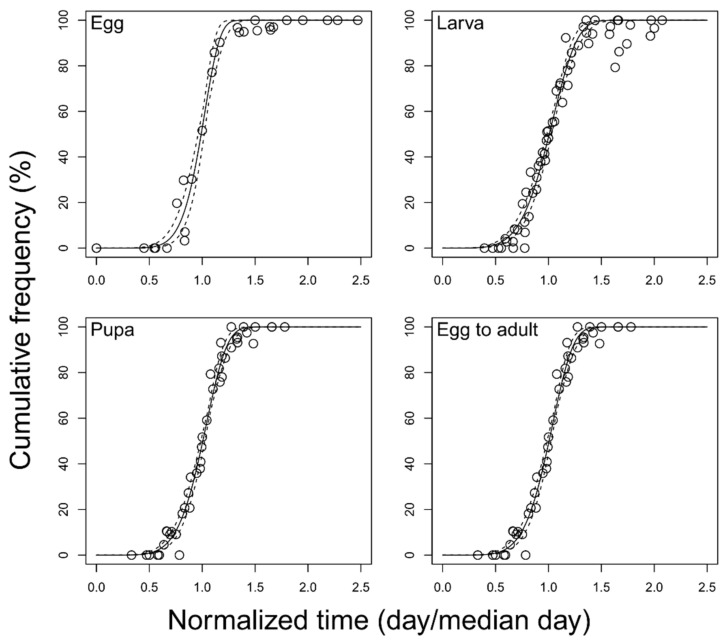
Cumulative distribution model for the frequency of development completion of *Feltiella acarisuga*. Estimated lines (solid lines) and 95% confidence interval (dashed lines).

All models describing the cumulative frequency (%) of development completion provided a good fit for each developmental stage (egg: *F* = 2363.3; df = 2, 24; *p* < 0.001; larva: *F* = 3211.0; df = 2, 57; *p* < 0.001; pupa: *F* = 3834.0; df = 2, 41; *p* < 0.001; total immature: *F* = 2926.5; df = 2, 53; *p* < 0.001) (Figure 4 and Table 6).

## 4. Discussion

In this study, we examined the development and survival of *F. acarisuga* at more broad temperature ranges than previous studies [23,24] to provide more elaborate information. Like other insect species, the temperature played a pivotal role in the development and survival of *F. acarisuga*. However, its performance in different temperatures varied according to the developmental stage. It was less sensitive to low temperatures, with survival in the order of eggs, pupae, and larvae (Figure 3). The eggs were able to hatch and develop successfully over a relatively wide temperature range as indicated by the $B_{80}$ values and lower development threshold.

The new findings in this study were the parameters of the non-linear curve for the development rate and survival of *F. acarisuga*. The response of insects to environmental factors can display unimodal curves [43], so a linear model cannot describe both extreme conditions at the same time. The convexity of the non-linear model can improve the limitations of the linear model and provide information that includes the operative range, extreme threshold, and optimum understanding of the lifecycle of the target insect [44]. The conceptual threshold temperatures for the development and survival models can be used to assess the possibility of establishment in specific conditions or a new region. The optimum and operative temperatures can be applied to predicting the emergence time and the viable number of individuals in both rearing and releasing conditions. However, there are limitations to be considered in the suggested non-linear model in this study. First, the upper thresholds of this study may not be a ‘true’ development threshold value. Since the observed lethal temperature of 35.4 °C was not used for curve fitting, the upper development thresholds are extrapolated values by the models. Even if the conformances of final models were evaluated (i.e., goodness of fit and relevance to observed lethal temperature), there is possibility that the estimated values are still not true. Therefore, our models may be improved through further study of development between 31.9 °C and 35.4 °C. The second limitation stems from the fact that our experiments were conducted under constant temperature. Experiments under fluctuating temperature can help estimate more realistic parameters associated with the development of target insects [45,46]. These parameters would be more useful in predicting natural occurrence in field conditions, and in optimizing rearing conditions suggested by our models [45,46].

Overall, the development rate was better at a relatively high-temperature range, but the survival was not (Figure 3). This means that there was a trade-off between the increase in the development rate and the decrease in the survival rate. Thus, there is no best point to pursue both, but the interval can be determined. The overlapping temperature range was estimated at 23.7–32.4, 24.3–29.2, 25.1–30.7, and 24.5–28.7 for rearing eggs, larvae, pupae, and the total immature stages, respectively. In a given range, the breeder can control the production rate while maintaining the survival rate (>80% of the maximum) by manipulating the temperature. Stage-specific emergence time (day or DD) distribution can be simulated by the cumulative distribution model and the development time at a given temperature. Conversely, temperatures outside of the estimated operative range would not be recommended for rearing and releasing. Temperatures around the potential lower ($T_{1}$) and upper ($T_{2}$) developmental threshold of the developmental curve would be avoided in ABC programs.

The total immature lower development threshold was estimated to be 8.2 °C in this study, 8.4 °C in the study by Gillespie et al. [24], and 3.2 °C in the study by Kim et al. [23]. These variations may have been attributed to experimental conditions, such as the sampling point (tested temperature), sample size (number of tested individuals), prey quality, and interaction with other factors. For instance, Gillespie et al. [24] reported the importance of high RH in the development and survival of *F. acarisuga*. It is approximately consistent with the results of this study from testing at more than 90% RH. In contrast, the results of Kim [23], from testing at 65% RH, showed much longer development times and higher mortality.

Although variations in *F. acarisuga* development existed in the rearing conditions, our results are preferable for mass-rearing programs because the development times in our study were the shortest overall compared to other studies [14,23,24,25]. For breeders, it would be better to choose the rearing conditions showing the best performance (i.e., development time) aside from the temperature effect. In addition, we tested with more insects at tighter temperature intervals, which could help reduce uncertainty resulting from the statistical model.

## 5. Conclusions

In summary, the findings in this study provide ecological information for the lifecycle of *F. acarisuga*, one of the important biological agents for controlling *T. urticae*. This information can help with effective rearing and establishment based on improved thermal response models. The results will contribute to the successful augmentative release of *F. acarisuga*.

## Figures and Tables

**Table 1:** Development time (d) (mean ± SE) in days and percent survival (s) of *Feltiella acarisuga* at constant temperatures (Temp).

Temp (°C)	Egg			Larval			Pupal			Egg to Adult		
	n ^1^	d	s	n	d	s	n	d	s	n	d	s ^4^
11.5	68	6.6 ± 0.10a ^2^	91.2	79	31.8 ± 2.05a	36.7	29	17.8 ± 0.71a	75.9	79	53.4 ± 1.88a	25.4
15.7	99	4.2 ± 0.07b	100.0	72	13.8 ± 0.56b	50.0	36	10.7 ± 0.22b	80.6	72	28.9 ± 0.59b	40.3
19.8	84	2.8 ± 0.06c	90.5	69	9.5 ± 0.28c	50.7	35	6.5 ± 0.26c	82.9	69	19.0 ± 0.27c	38.0
23.4	36	2.3 ± 0.09d	97.2	84	5.7 ± 0.22d	58.3	49	4.7 ± 0.14d	79.6	84	12.4 ± 0.27d	45.1
27.7	72	1.8 ± 0.06e	91.7	82	4.8 ± 0.14d	61.0	50	4.0 ± 0.14d	82.0	82	10.5 ± 0.16d	45.8
31.9	53	1.7 ± 0.08e	88.7	70	5.7 ± 0.12d	37.1	26	3.5 ± 0.18d	73.1	70	10.1 ± 0.24d	24.1
35.4	139	- ^3^	0.0	98	-	0.0	72	-	0.0	98	-	0.0

^1^ Initial number for treatment ^2^ Means followed by the same letter within a column are not significantly different (Tukey’s HSD; *p* > 0.05) ^3^ No individual survived ^4^ Calculated by multiplying the survival rates of eggs and larvae to adults.

**Table 2:** Estimated parameter values of the linear development rate model for *Feltiella acarisuga*.

Stage	Parameter (Mean ± SE)		Lower Development Threshold (°C)	Thermal Constant (DD)
	**a**	**b**		
Egg	0.024 ± 0.0007	−0.131 ± 0.0158	5.5	41.7
Larval	0.011 ± 0.0010	−0.105 ± 0.0207	9.5	90.9
Pupal	0.012 ± 0.0007	−0.083 ± 0.0154	6.9	83.3
Egg to adult	0.005 ± 0.0004	−0.041 ± 0.0075	8.2	200.0

**Table 3:** Assessment of non-linear models based on the residual sum of squares (RSS) and conceptual upper development threshold ($T_{2}$).

Model	$\mathrm{RSS}{\;}^{1}/T_{2}{\;}^{2}$				Average Rank	Final Selection
	Egg	Larval	Pupal	Egg to Adult		
LRF	2533.6/43.4	23.7/35.8	705.2/44.5	50.9/39.8	4.5/1.8	
Logan-6	67.8/35.4	5.1/35.0	21.2/35.0	1.4/35.1	2.5/1.0	Selected
Lactin-2	2582.8/41.3	2.9/34.9	699.6/41.5	52.8/37.7	4.0/1.8	
Performance-2	1228/36.9	4.9/33.4	631.0/38.7	0.6/35.1	2.5/1.5	
Beta	2842.8/43.8	2.5/35.3	780.5/45.1	51.2/38.9	4.5/1.8	
Briere-2	2219.7/37.1	3.4/32.3	759.3/39.9	0.4/33.9	3.0/1.5	

^1^ RSS multiplied by 10^5^ ^2^ Temperature (°C) at which development rate is zero in the range after the optimal temperature.

**Table 4:** Estimated parameter values of the six non-linear models describing the relationship between temperature and development rate of *Feltiella acarisuga*.

Model	Parameter	Egg	Larval	Pupal	Egg to Adult
LRF	$\mu_{opt}$	9.26 × 10^−7^	0.13 × 10^−^^7^	5.22 × 10^−^^7^	3.54 × 10^−^^7^
	$T_{opt}$	19.0488	18.6848	20.7438	19.7650
	$T_{1}$	−5.3358	1.5729	−2.9197	−0.3839
	$T_{2}$	43.4274	35.791	44.4095	39.8056
Logan-6	ψ	0.1021	0.0433	0.0236	0.0087
	ρ	0.1496	0.1671	0.1774	0.1625
	$T_{u}$	35.4276	35.0051	35.0338	35.0770
	ΔT	6.1817	5.7561	5.2619	5.5134
Lactin-2	ρ	0.0851	0.1544	0.0939	0.1249
	$T_{u}$	42.7528	35.2245	42.7897	38.1585
	ΔT	11.0830	6.4580	10.4435	7.9811
	λ	−0.2446	−0.0312	−0.1136	−0.0221
Performance-2	c	0.0252	0.0116	0.0131	0.0050
	$T_{1}$	5.6686	9.2970	7.7346	8.3325
	K	0.3978	0.7557	0.3533	0.5603
	$T_{2}$	36.9831	33.3749	38.7301	35.1266
Beta	$r_{m}$	0.5735	0.2142	0.2879	0.1012
	$T_{2}$	43.8128	35.3243	45.0534	38.8855
	$T_{m}$	31.6761	28.6001	32.9301	30.1988
	$T_{1}$	−8.1044	−18.4536	−2.4553	−10.7691
Briere-2	α	2.88 × 10^−^^4^	2.58 × 10^−^^4^	1.22 × 10^−^^4^	0.93 × 10^−^^4^
	$T_{1}$	−2.6248	6.3264	3.5766	4.1890
	$T_{2}$	37.1227	32.3286	39.9349	33.9764
	β	2.7946	4.6746	2.1764	3.9169

**Table 5:** Estimated parameter values of the temperature-dependent survival model for *Feltiella acarisuga*.

Stage	Parameter		
	a (Mean ± SE)	b (Mean ± SE)	c (Mean ± SE)
Egg	8.439 ± 0.5296	−0.805 ± 0.1101	0.020 ± 0.0027
Larval	5.956 ± 0.3888	−0.208 ± 0.0369	0.005 ± 0.0008
Pupal	7.665 ± 0.3879	−0.501 ± 0.0584	0.012 ± 0.0014
Egg to adult	5.508 ± 0.2488	−0.140 ± 0.0233	0.003 ± 0.0005

**Table 6:** Estimated parameter values of development distribution model for *Feltiella acarisuga*.

Stage	Parameter	
	α (Mean ± SE)	β (Mean ± SE)
Egg	1.038 ± 0.0108	7.839 ± 0.7049
Larval	1.078 ± 0.0082	5.433 ± 0.2982
Pupal	1.066 ± 0.0065	6.242 ± 0.3050
Egg to adult	1.039 ± 0.0043	10.534 ± 0.6176

## Data Availability

The experimental data are presented in “Table 1”. Other data in this study are available on request from the corresponding authors.
